# Lapatinib sensitivity in nasopharyngeal carcinoma is modulated by SIRT2-mediated FOXO3 deacetylation

**DOI:** 10.1186/s12885-019-6308-7

**Published:** 2019-11-14

**Authors:** Sathid Aimjongjun, Zimam Mahmud, Yannasittha Jiramongkol, Glowi Alasiri, Shang Yao, Ernesto Yagüe, Tavan Janvilisri, Eric W.-F. Lam

**Affiliations:** 1 Department of Surgery and Cancer, Imperial College London, Hammersmith Hospital Campus, London, W12 0NN UK; 2 Graduate Program in Molecular Medicine, Multidisciplinary Unit, Faculty of Science, Mahidol University, Bangkok, Thailand; 3 Department of Biochemistry, Faculty of Science, Mahidol University, Bangkok, Thailand

**Keywords:** Nasopharyngeal carcinoma, Sirtuin, FOXO3, Chemoresistance, Lapatinib, Acetylation

## Abstract

**Background:**

Chemoresistance is an obstacle to the successful treatment of nasopharyngeal carcinoma (NPC). Lapatinib is a targeted tyrosine kinase inhibitor therapeutic drug also used to treat NPC, but high doses are often required to achieve a result. To investigate the mechanism for the development of Lapatinib resistance, we characterised a number of NPC cell lines to determine the role of FOXO3 and sirtuins in regulating NPC resistance.

**Methods:**

Sulforhodamine B (SRB) assays, Clonogenic assays, Protein extraction, quantification and western blotting, RT qPCR, Co-immunoprecipitation assay.

**Results:**

To explore novel treatment strategies, we first characterized the Lapatinib-sensitivity of a panel of NPC cell lines by SRB and clonogenic cytotoxic assays and found that the metastatic NPC (C666–1 and 5-8F) cells are highly resistant whereas the poorly metastatic lines (6-10B, TW01 and HK-1) are sensitive to Lapatinib. Western blot analysis of the Lapatinib-sensitive 6-10B and resistant 5-8F NPC cells showed that the expression of phosphorylated/inactive FOXO3 (P-FOXO3;T32), its target FOXM1 and its regulator SIRT2 correlate negatively with Lapatinib response and sensitivity, suggesting that SIRT2 mediates FOXO3 deacetylation to promote Lapatinib resistance. In agreement, clonogenic cytotoxic assays using wild-type and *foxo1/3/4*^*−/−*^ mouse embryonic fibroblasts (MEFs) showed that FOXO1/3/4-deletion significantly attenuates Lapatinib-induced cytotoxicity, confirming that FOXO proteins are essential for mediating Lapatinib response. SRB cell viability assays using chemical SIRT inhibitors (i.e. sirtinol, Ex527, AGK2 and AK1) revealed that all SIRT inhibitors can reduce NPC cell viability, but only the SIRT2-specific inhibitors AK1 and AGK2 further enhance the Lapatinib cytotoxicity. Consistently, clonogenic assays demonstrated that the SIRT2 inhibitors AK1 and AGK2 as well as SIRT2-knockdown increase Lapatinib cytotoxicity further in both the sensitive and resistant NPC cells. Co-immunoprecipitation studies showed that besides Lapatinib treatment, SIRT2-pharmaceutical inhibition and silencing also led to an increase in FOXO3 acetylation. Importantly, SIRT2 inhibition and depletion further enhanced Lapatinib-mediated FOXO3-acetylation in NPC cells.

**Conclusion:**

Collectively, our results suggest the involvement of SIRT2-mediated FOXO3 deacetylation in Lapatinib response and sensitivity, and that SIRT2 can specifically antagonise the cytotoxicity of Lapatinib through mediating FOXO3 deacetylation in both sensitive and resistant NPC cells. The present findings also propose that SIRT2 can be an important biomarker for metastatic and Lapatinib resistant NPC and that targeting the SIRT2-FOXO3 axis may provide novel strategies for treating NPC and for overcoming chemoresistance.

## Background

Nasopharyngeal carcinoma (NPC) is a malignancy of head and neck epithelial cells that originates from the nasopharyngeal cavity. NPC is an important health problem in Southern China and Southeast Asia [1], with about 86,500 NPC new cases recorded annually, and over 40,000 die from the disease every year in Asia alone [2]. The most common treatment for NPC is the combination of radiotherapy and chemotherapy. Radiotherapy is a mainstay for treatment for early NPC, while chemotherapy is frequently used for the advanced locoregionally NPC. It has been shown that the combination of chemotherapy and radiation enhances the survival rates [3]. However, advanced NPCs usually fail to respond to chemotherapy treatment because of the development of drug resistance that leads to metastasis and relapse, resulting in poor prognosis [4]. In consequence, there is an urgent need for the identification of novel therapeutic strategies as well as the mechanisms of chemotherapeutic resistance for NPC. To date, the mechanisms for the development of resistance to conventional therapeutics, such as cisplatin (CDDP), paclitaxel (PTX), 5-fluorouracil (5-FU), and vincristine (VCR), for NPC have been extensively studied [5–8]. These include overexpression of multidrug-resistant genes, enhanced DNA repair, deregulation of apoptosis, and/or modifications of drug targets [5–8].

Both the epidermal growth factor receptor (EGFR) and HER2 (human epidermal growth factor receptor 2; ERRB2) are commonly overexpressed in NPC [9, 10]. Moreover, activation of EGFR/HER2 pathway has also been shown to promote NPC progression and invasion [11]. Previous studies have demonstrated that the dual EGFR/HER2 tyrosine kinase inhibitor, Lapatinib, can restrict cell proliferation and invasion, and promote anoikis in NPC cells [12, 13]. Despite its promising efficacy, Lapatinib has a half maximal inhibitory concentration (IC_50_) in the micromolar range in most cell lines [13], and strategies to sensitize NPC to Lapatinib are currently under investigation.

FOXO3 and FOXM1 are members of the forkhead box transcription factor family which play opposite roles in tumorigenesis, drug resistance and cancer progression. FOXO3 is a key tumour suppressor which functions downstream of the PI3K/AKT (protein kinase B) signalling pathway. Conversely, FOXM1 is an important oncogene that promotes cell transformation, cancer progression and resistance to chemotherapy. In addition, FOXM1 and FOXO3 negatively regulate the expression of one another and compete for same binding sites to promote or inhibit the transcription of target genes [14]. Furthermore, FOXO3 has also be shown to be regulated by post-translational modifications, such as phosphorylation, methylation, ubiquitination, glycosylation and acetylation. The reversible acetylation/deacetylation of FOXO3 is regulated by class I histone deacetylases (HDACs) as well as class III HDACs named sirtuins (SIRT 1–7) and histone acetyl transferases (HATs e.g. p300/CBP) [15]. The function of SIRT proteins is to reverse the acetylation of FOXO3 to induce the cell cycle arrest, protection from oxidative stress and to repress the expression of apoptotic genes [16]. In NPC cells, FOXM1 is overexpressed and it is associated with cancer metastasis and chemoresistance [17, 18]. In NPC patients, low FOXO3 and high HIF-1 expression has been found to be correlated with poor prognosis in NPC [19]. However, the role and regulation of FOXO3 and FOXM1 in NPC have not been not fully investigated.

In this study, we investigated the mechanism of action of a dual tyrosine kinase inhibitor Lapatinib using high and low metastatic NPC cells and found that the deacetylation of FOXO3 by SIRT2 plays an important role in the regulation of Lapatinib response and resistance in NPCs.

## Methods

### Cell culture

The NPC cell line TW01 is an established cell line derived from moderately differentiated keratinising NPC tissues, and was kindly provided by Prof. Chin-Tarng Lin, National Taiwan University, Taipei [20]. TW01 cells were cultured with DMEM supplemented with 10% v/v foetal bovine serum (FBS), 100 U/mL penicillin, and 100 μg/mL streptomycin. Poorly differentiated human NPC cell lines SUNE 5–8 F (highly tumorigenic and metastatic) and 6-10B (highly tumorigenic, but poorly metastatic) derived from the parental line SUNE-1, were obtained from the Prof. Qingling Zhang, Southern Medical University, Guangzhou China [21, 22]. HK1, a well-differentiated squamous carcinoma line was obtained from the Queen Elizabeth Hospital, University of Hong Kong [23]. C666–1 is an undifferentiated NPC cell line obtained from Prof. Maria L. Lung, Center for Nasopharyngeal Carcinoma Research, University of Hong Kong [24]. These last four NPC cell lines were cultured in RPMI supplemented with 10% FBS, 100 U/mL penicillin, and 100 μg/mL streptomycin. All NPC cells were maintained in a humidified incubator with 10% CO_2_ at 37 °C. Wild type mouse embryonic fibroblasts (MEFs) and triple knock-out *foxo1/3/4*^*−/−*^ MEFs were kind gifts from Prof. Boudewijn Burgering, UMC, Utrecht, the Netherlands, and have been described previously [25]. MEF cells were cultured in Dulbecco’s modified eagle’s medium (DMEM) (Sigma Aldrich, Poole, UK) and supplemented with 10% (v/v) foetal calf serum (FCS) (First Link Ltd., Birmingham, UK), 100 Unit/ml penicillin/streptomycin (Sigma-Aldrich, UK) and 2 mM glutamine and maintained at 37 °C in a humidified atmosphere containing 10% CO_2_. All cell lines were subjected to DNA fingerprinting analysis using the AmpF/STR Identifiler PCR Amplification Kit (Applied Biosystems, Foster City, USA) and are free from mycoplasma contamination.

### siRNA mediated gene knockdown

For gene knockdown, cells were plated in at 60–70% confluency. The following day, cells were transfected with ON-TARGET plus siRNA smart pools (GE Dharmacon) targeting SIRT2 (L^− 004826^-00-0005) using oligofectamine (Invitrogen, UK) according to the manufacturer’s protocol. Non-Targeting siRNA pool (GE Dharmacon; D-001210-01-05) was used as transfection control.

### Sulforhodamine B colorimetric assay

A total of 1000 NPC cells per well were seeded in a 96-wells plate. One day after seeding, NPC cells were treated with increasing concentrations of Lapatinib for 24 and 48 h. The cells were fixed with 40% trichloroacetic acid at 4 °C for 1 h, washed 3 times with PBS and stained with 0.4% (w/v) sulforhodamine B (SRB) solution at room temperature for 1 h. Following the staining, the cells were washed 5 times with 1% acetic acid and air-dried overnight. The protein bound dye was dissolved in 10 mM Tris base solution and the absorbance was measured at 492 nm using a microplate reader (Sunrise, Tecan; Männedorf, Switzerland).

### Clonogenic assay

A total of 2000–10,000 cells were seeded into 6-well plates and incubated overnight. The cells were then treated for 72 h with varying concentrations of Lapatinib and SIRT inhibitor (SIRT-i). DMSO (Sigma-Aldrich,) was used as a vehicle and blank. The drug was removed and surviving cells were left to form colonies. After 1–2 weeks of incubation, colonies were fixed with 4% paraformaldehyde for 15 min at room temperature and then washed with PBS three times. Crystal violet (0.5% w/v) was used to stain the fixed cells for 30 min, following which the plates were washed with tap water. Plates were then left to dry overnight. Quantification was achieved by solubilising dye with 33% acetic acid and the absorbance was measured at 592 nm using Tecan Microplate reader.

### Western blotting and antibodies

Western blotting was performed with whole cell extracts prepared by lysing cells with NP40 lysis buffer [1% NP40, 100 mmol/L NaCl, 20 mmol/L Tris-HCl (pH 7.4), 10 mmol/L NaF, 1 mmol/L Na orthovanadate, 30 mmol/L Na β-glycerophosphate, and protease inhibitors (“Complete” protease inhibitor mixture, as instructed by the manufacturer, Roche Applied Science)] on ice for 15 min. After incubation, the lysates were centrifuged at 13,000×g for 10 min, and the supernatant was collected. Protein concentrations were determined using a BCA protein assay kit (ThermoFisher Scientific, Waltham, MA, USA). Ten micrograms of protein were size fractionated using SDS-PAGE and electro-transferred onto nitrocellulose membranes (BioRad, San Diego, CA, USA). Membranes were blocked in 5% (w/v) bovine serum albumin (BSA) in TBS plus 0.5% (v/v) Tween for 1 h at room temperature and then incubated with specific antibodies overnight at 4 °C. The antibodies against EGFR, P-EGFR, ERBB2, P-ERBB2, ERBB3, P-ERBB3, JNK, P-JNK, p38, P-p38, c-Myc, p27^Kip1^, P-FOXO3 (T32), FOXO3, cyclin B1, P-AKT (S473), AKT and β-Tubulin were purchased from Cell Signalling Technology (New England Biolabs Ltd., Hitchin, UK). The antibody against FOXM1 (C^− 20^) was purchased from Santa Cruz Biotechnology (Santa Cruz, CA, USA). Primary antibodies were detected using horseradish peroxidase-linked anti-mouse or anti-rabbit conjugates (Dako, Glostrup, Denmark) and visualized using the ECL detection system (Perkin Elmer, Beaconsfield, UK).

### Co-Immunoprecipitation assay

NPC cell lysates were prepared in IP buffer (1% Nonidet P^− 40^, 150 mM NaCl, 50 mM Tris-HCl [pH 7.4], 10 mM NaF, 1 mM Na_3_VO_4_, 10 mM N-ethyl-amide (NEM) and protease inhibitors [Complete protease inhibitor cocktail; Roche, Lewes, UK]). The pre-cleared lysate was immunoprecipitated with the indicated antibodies and with protein A/G-sepharose for 4 h. After that, the fractionated supernatant was transferred to the new tube. Samples and incubated overnight with 0.5 μg of primary antibody-FOXO3 (sc-11,351, Santa Cruz) [1, 26], Anti-acetyl lysine (Cell-Signalling technology) or IgG negative control (2729, Cell-Signalling technology) (1500) at 4 °C on a rotator. The supernatant was discarded on the following day and beads were washed three times with PBS. Samples were boiled with 2 × SDS sample buffer for 5 min at 100 °C and were analysed by SDS-PAGE followed by western blotting.

### Gene expression analysis by RT-qPCR

Total mRNA was extracted from cell pellets using the RNeasy Mini Kit (Qiagen, UK) according to manufacturer’s instructions. The purity and concentration of mRNA samples were determined by measuring the spectrophotometric absorption at 260 nm and 280 nm using a NanoDrop ND-1000. Then, 2 μg of the extracted total RNA was used as a template for first strand cDNA synthesis reaction, The resulting first-strand cDNA was then used as template in the real-time PCR. Real time quantitative PCR (RT-qPCR) was performed on 100 ng of cDNA with SYBER-Green Master Mix (Applied BioSystems). All RT-qPCR assays were assayed in 96-well plates in the ABI PRISM® 7900 HT Fast Real-time PCR System (Applied BioSystems) on a cycling program of 90 °C for 10 min for enzyme activation followed by 40 cycles of denaturation and primer annealing/extension consisting of 95 °C for 3 s and 60 °C for 30s respectively. The nonregulated ribosomal housekeeping gene, *RPL19* was used as an internal control to normalize gene expression between samples. All samples were done in triplicates. Primer sequences used for RT-qPCR are,

RPL19-F: 5’GGTGCTTCCGATTCCAGAGT3’,

RPL19-R: 5’CCCATTCCCTGATCGCTTGA3’,

Erbb2-F: 5’GCTCTTTGAGGACAAGTATG3’,

Erbb2-R: 5’AAGATCTCTGTGAGACTTCG3’,

Egfr-F: 5’CTGTCGCAAAGTTTGTAATG3’,

Egfr-R: 5’GAATTTCTAGTTCTCGTGGG3’,

Foxo3-F: 5’CCGGACAAACGGCTCACT3’,

Foxo3-R: 5’GGCACACAGCGCACCAT3’,

Foxo4-F:5’AGGACAAGGGTGACAGCAAC3’,

Foxo4-R: 5’GGTTCAGCATCCACCAAGAG3’,

Foxo1-F: 5’AAGAGCGTGCCCTACTT-CAA3’,

Foxo1-R: 5’TCCTTCA-TTCTGCACTCGAA 3′.

### Statistical data analysis

The results were represented as the means ± SEM of at least 3 separate experiments. For categorical variables, we used the analysis of variance (ANOVA) which was followed by a Bonferroni’s post-hoc test for multiple comparisons. Student’s t test was used to analyse the statistical significance of differences between the control groups and the experimental groups. The differences were considered as significant at *p* < 0.05. The GraphPad Prism 5.0 software (version 5.00 for Windows, GraphPad Software; San Diego, California, USA) was used for the statistical analyses. ImageJ software was used to analyse and compare the intensity of the protein bands obtained from Western blots.

## Results

### Effects of Lapatinib on cell viability of different nasopharyngeal carcinoma (NPC) cell lines

**Fig. 1:**
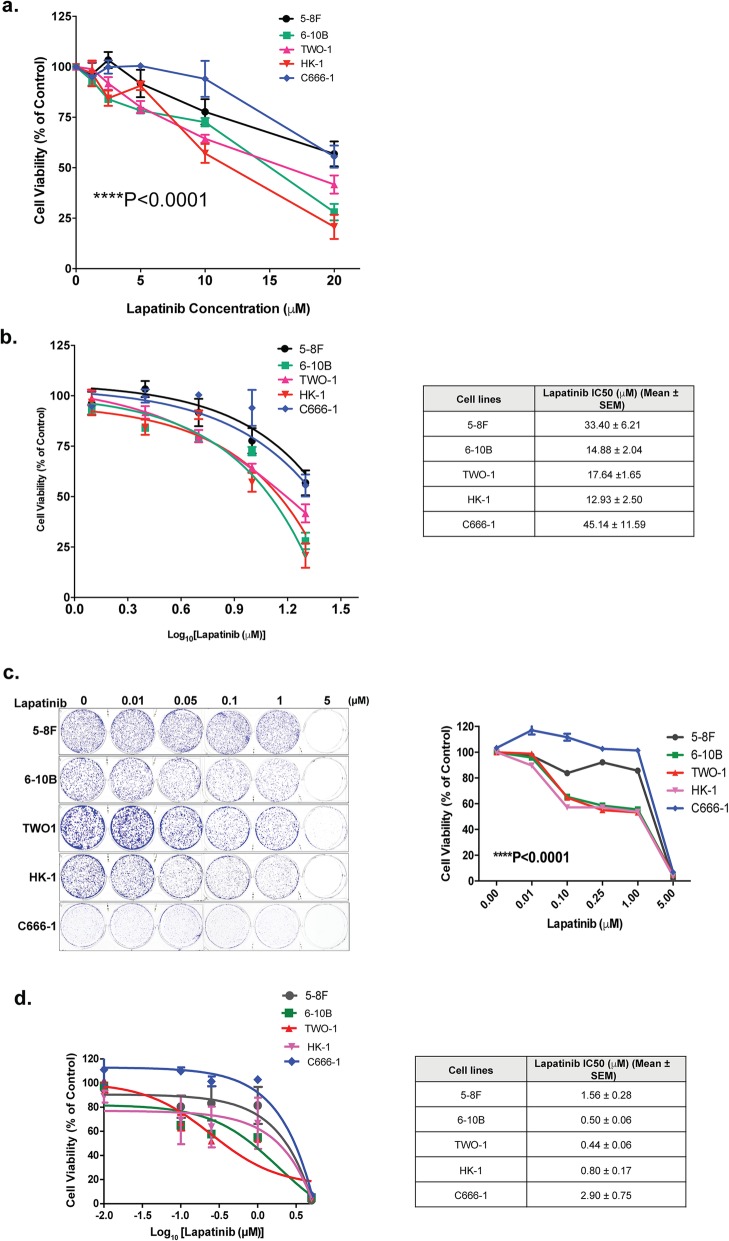
Effects of Lapatinib on cell growth and proliferation of NPC cells. **a** Lapatinib sensitivity curves in NPC cells was determined by sulforhodamine B staining. Cells (3 × 10^3^ / well) were seeded in 96-well plates and treated with increasing concentrations of Lapatinib for 72 h before SRB staining and measurement of optical density at 492 nm. **b** Best-fit curves were generated to determine IC_50_ for each NPC cell lines against Lapatinib. **c** NPC cells were seeded into 6-well plates (1 × 10^3^ cells / well) and treated with Lapatinib for 72 h. Colony formation was monitored after 15 days, stained with crystal violet (representative images are shown) and absorbance at 592 nm determined (right panel). **d** Best-fit curves were generated to determine IC_50_ for each NPC cells against Lapatinib in terms of clonogenic assay. Numerical data represent the average ± SEM of three independent experiments (**** *P* < 0.0001)

In order to identify the cellular mechanisms which modulate Lapatinib sensitivity, we first analysed its effect on the proliferation of NPC cell lines with distinct metastatic status. To this end, two highly metastatic (C666–1 and 5-8F) and three poorly metastatic (6-10B, TW01 and HK-1) NPC cell lines were treated with increasing concentrations of Lapatinib (from 0 μM to 20 μM) for 48 h and their viability measured by the sulforhodamine B (SRB) assay. The results showed that Lapatinib caused a dose-dependent reduction of cell proliferation in all NPC cells. The results also indicated that there were significant differences in Lapatinib sensitivity between the NPC cell lines at 48 h (Fig. 1a) (2-way ANOVA, ****P < 0.0001). Moreover, the highly metastatic (C666–1 and 5-8F) lines were significantly more resistant to Lapatinib than the three poorly metastatic lines (6-10B, TW01 and HK-1) (Fig. 1b). These results were further confirmed by clonogenic assays, in which the surviving cells were allowed to further proliferate and form clones. The highly metastatic C666–1 and 5-8F lines were also significantly more resistant to Lapatinib than the three poorly metastatic lines (6-10B, TW01 and HK-1) in longer term assays (Fig. 1c, d).

### Effects of Lapatinib on the expression and activity of proteins involved in EGFR/ERBB2 signalling in the SUNE 5-8F and 6-10B NPC cells

**Fig. 2:**
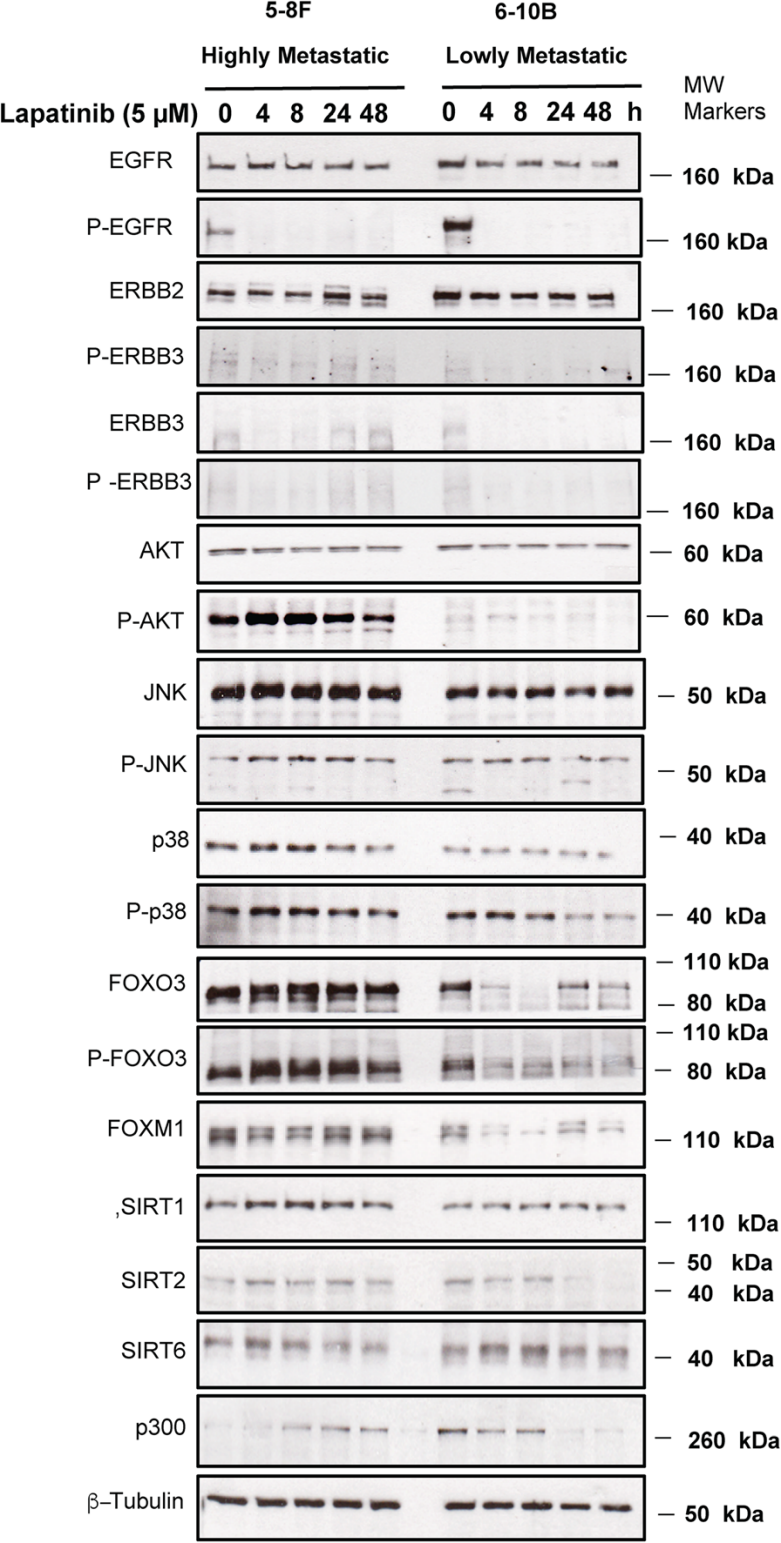
Effects of Lapatinib on the expression and activity of proteins involved in EGFR/ERBB2 signalling pathways in high and low metastasis NPC cell lines. The high 5-8F and low 6-10B metastatic NPC cells were treated with 5 μM Lapatinib for 0. 4, 8, 24, 48 h. Protein lysates from whole-cell extracts were collected and then analysed by western blotting using the antibodies against ERBB1, P-ERBB1, ERBB2, P-ERBB2, ERBB3, P-ERBB3, AKT, P-AKT, SIRT1, SIRT2, SIRT6, p300, JNK, P-JNK, p38, P-p38, FOXO3, P-FOXO3(T32), FOXM1, and β-tubulin. Molecular markers are shown on the right panel of each blot. Representative results of three repeats are shown. Protein levels were determined by using ImageJ and the protein levels relative to β-tubulin are shown below protein bands as ratios to the 0 h Lapatinib controls

In order to identify the potential mechanisms involved in modulating Lapatinib sensitivity, we analysed by Western blotting the effects of Lapatinib on the expression of molecules implicated in Lapatinib signalling and sensitivity. To this end, the two NPC cell lines, SUNE 5-8F (highly metastatic) and 6-10B (poorly metastatic) with differential Lapatinib sensitivity (low and high, respectively) were treated with 5 μM Lapatinib for 0 to 48 h (Fig. 2). Western blot results showed a reduction in P-ERBB1 and P-ERBB2 upon Lapatinib treatment (Fig. 2), indicating the drug is effective in inhibiting ERBB1/ERBB2 activity in both NPC lines. Notably, both total ERBB1 and ERBB2, but not ERBB3, were expressed at high levels in both NPC lines and their expression remained at high levels after Lapatinib treatment. FOXO3 has previously been shown to mediate the cytotoxic function of Lapatinib through repressing FOXM1 expression. The p38 and Jun N-terminal kinase (JNK) MAPKs have also been demonstrated to phosphorylate and activate FOXO3 in response to Lapatinib, while AKT has been reported to be repressed by Lapatinib, leading to FOXO3 dephosphorylation (T32) and derepression. In agreement, western blot analysis showed that FOXO3 became dephoshorylated (T32) and activated upon Lapatinib treatment in the sensitive 6-10B, but remained phosphorylated (T32) and inactivated in the resistant 5-8F cells. FOXO3 dephosphorylation was also correlated with downregulation of FOXM1 expression in 6-10B but not in 5-8F cells, further confirming the role of FOXO3 in mediating Lapatinib action. Interestingly, the induction of p38 and JNK phosphorylation was not observed in the sensitive 6-10B cells upon Lapatinib treatment, suggesting p38 and JNK are unlikely to be instrumental in activating FOXO3 in response to Lapatinib. FOXO3 activity can also be enhanced by acetylation, which have been shown to be promoted by EP300 and repressed by the nuclear sirtuins, SIRT1, − 2 and − 6. Western blot analysis showed EP300 was at low levels in the resistant, metastatic 5-8F cells but was induced upon Lapatinib treatment, whereas EP300 was expressed at high levels in 6-10B and was downregulated in response to Lapatinib. The results also showed SIRT1 and − 6 were expressed at comparable levels in both 5-8F and 6-10B cells, and their expression was not substantially affected by Lapatinib treatment. The observed expression patterns of EP300, SIRT1 and SIRT6 suggested that they are unlikely to be responsible for FOXO3 activation by Lapatinib, even though EP300, SIRT1 and SIRT6 could still modulate FOXO3 acetylation and activity in these NPC cells. By contrast, upon Lapatinib treatment the expression levels of SIRT2 remained constitutively high in the resistant 5-8F cells, but were downregulated by Lapatinib in the sensitive 6-10B cells, suggesting that SIRT2 can potentially mediate Lapatinib response through modulating FOXO3 acetylation and activity in these NPC cells. These results suggest that highly metastatic and EBV (Epstein Barr virus)-positive cell lines are more resistant to Lapatinib, probably due to their high levels of FOXM1 and P-FOXO3 expression, suggesting that SIRT2 induces Lapatinib resistance through FOXO3 acetylation and activity, which in turn led to reduction in FOXO3 activity.

### FOXOs mediate the cytotoxic function of Lapatinib

**Fig. 3:**
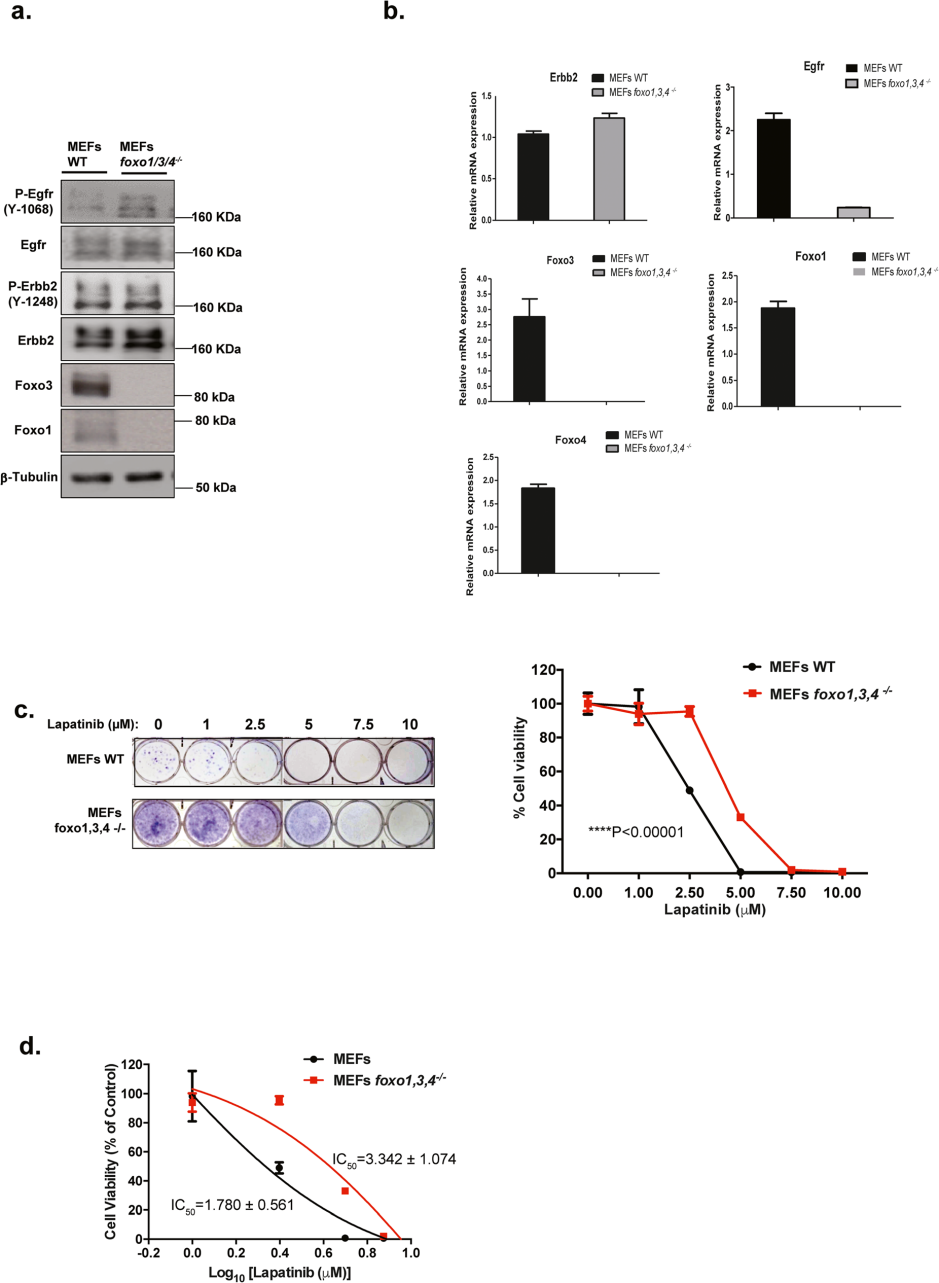
FOXOs regulate Lapatinib sensitivity in mouse embryonic fibroblasts. **a** Western blot analysis was performed to analyse the protein levels of P-EGFR, EGFR, P-ERBB2, ERBB2, FOXO3, FOXO1 in MEFs and *Foxo1,3,4*^−/−^ MEFs. Representative western blot results are shown. Tubulin was used as a protein loading control. **b** The mRNA levels of *EGFR, ERBB2, FOXO3, FOXO1* and *FOXO4* were assessed by real-time qPCR. The *RPL19* housekeeping gene transcript was used as a control to normalise gene expression. **c** Wild-type (WT) MEFs and *Foxo1,3,4*^−/−^ MEFs were treated with increasing concentrations of Lapatinib for 3 days and colony formation was observed for 1 week. Cells were then stained with crystal violet and absorbance measured at 592 nm (right panel). The colony formation capacity was analysed by two-way ANOVA and found to be significantly different (***p < 0.00001) from one another. **d** Best-fit curves were generated to determine IC_50_ for each cell line against Lapatinib in terms of clonogenicity. Numerical data represent the average ± SEM of three different experiments (**** *P* < 0.0001)

FOXO proteins have previously been shown to be functionally redundant and can compensate for one another [27]. To verify the role of FOXO3 in mediating the cytotoxic response of Lapatinib, the effects of Lapatinib treatment were investigated in wild-type (WT) and *foxo1/3/4* ^−/−^ MEFs. Western blot and RTq-PCR analyses confirmed the Foxo1/3/4 knockout in the *foxo1/3/4* ^−/−^ MEFs and demonstrated EGFR/HER2 overexpression in both wild-type (WT) and *foxo1/3/4* ^−/−^ MEFs (Fig. 3a, b), implying that they are potentially sensitive to Lapatinib inhibition. Clonogenic assays showed that both wild-type (WT) and *foxo1/3/4* ^−/−^ MEFs are indeed sensitive to the antiproliferative functions of Lapatinib (Fig. 3c). Clonogenic assays also revealed that *foxo1/3/4-*deficient MEFs displayed higher self-renewal ability and was significantly less sensitive to the antiproliferative effects of Lapatinib compared with the WT MEFs (2-way ANOVA, ****P < 0.0001) (Fig. 3c and d), confirming the key role of FOXOs in mediating the cytotoxic functions of Lapatinib.

### The clonogenic survival of NPC cells is sensitive to chemical inhibitors of SIRTs

**Fig. 4:**
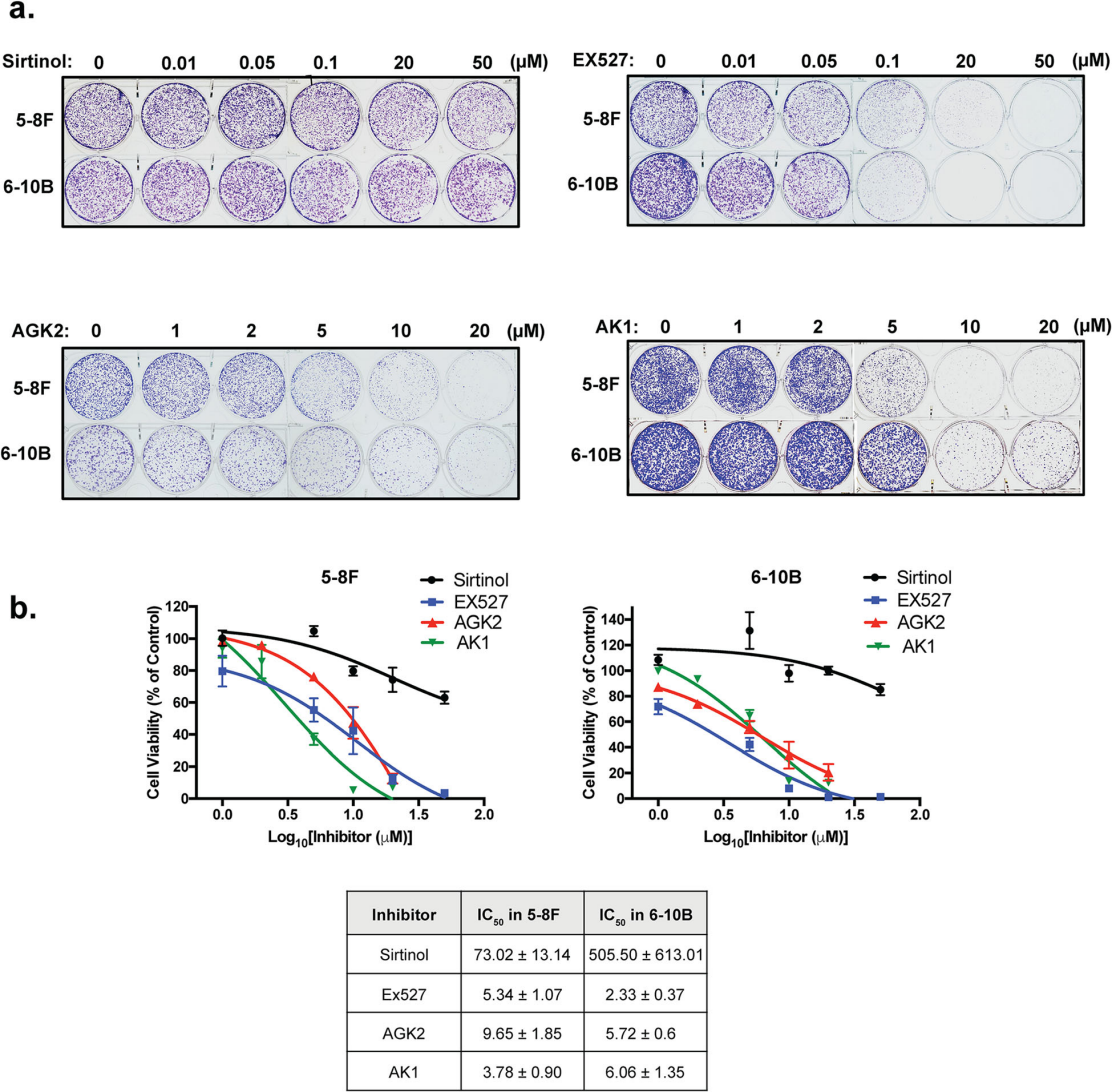
SIRT inhibitors limit long-term cell growth and colony formation capacity of high and low metastatic NPC cells. **a** 5-8F and 6-10B cells were seeded in 6-well plates and treated with the drug concentration indicated for 72 h. Then, cell culture media was removed and colony formation observed for 15 days. At the end of this time period, cells were fixed with 4% paraformaldehyde and stained with crystal violet. Representative pictures from triplicate experiments are shown. **b** The stain was solubilised with 33% acetic acid and absorbance at 592 nm were obtained. Best fit-curves were generated to determine the IC_50_ of each drug in 5-8F and 6-10B cells

The anti-proliferative activity of FOXO3 is promoted by acetylation which can be attenuated by nuclear SIRTs. In order to investigate if the viability of the NPC cells are also modulated by SIRTs, we subjected both the Lapatinib resistant 5-8F and sensitive 6-10B NPC cells to treatment with the SIRT1-specific (ie. EX527), SIRT2-specific (ie. AK1 and AGK2) and pan-SIRT (ie. Sirtinol) inhibitors individually for 72 h and their subsequent long-term survival examined by clonogenic assays (Fig. 4a and b). The results showed that all four SIRT-inhibitors decreased the clonogenic survival of both NPC cell lines, with EX527, AK1 and AGK2 being more effective compared to Sirtinol, suggesting that the activity of SIRT1 and − 2 might have a role in modulating the long-term viability of NPC cells.

### SIRT2 inhibitors AK1 and AGK2 function cooperatively with Lapatinib in NPC cells

**Fig. 5:**
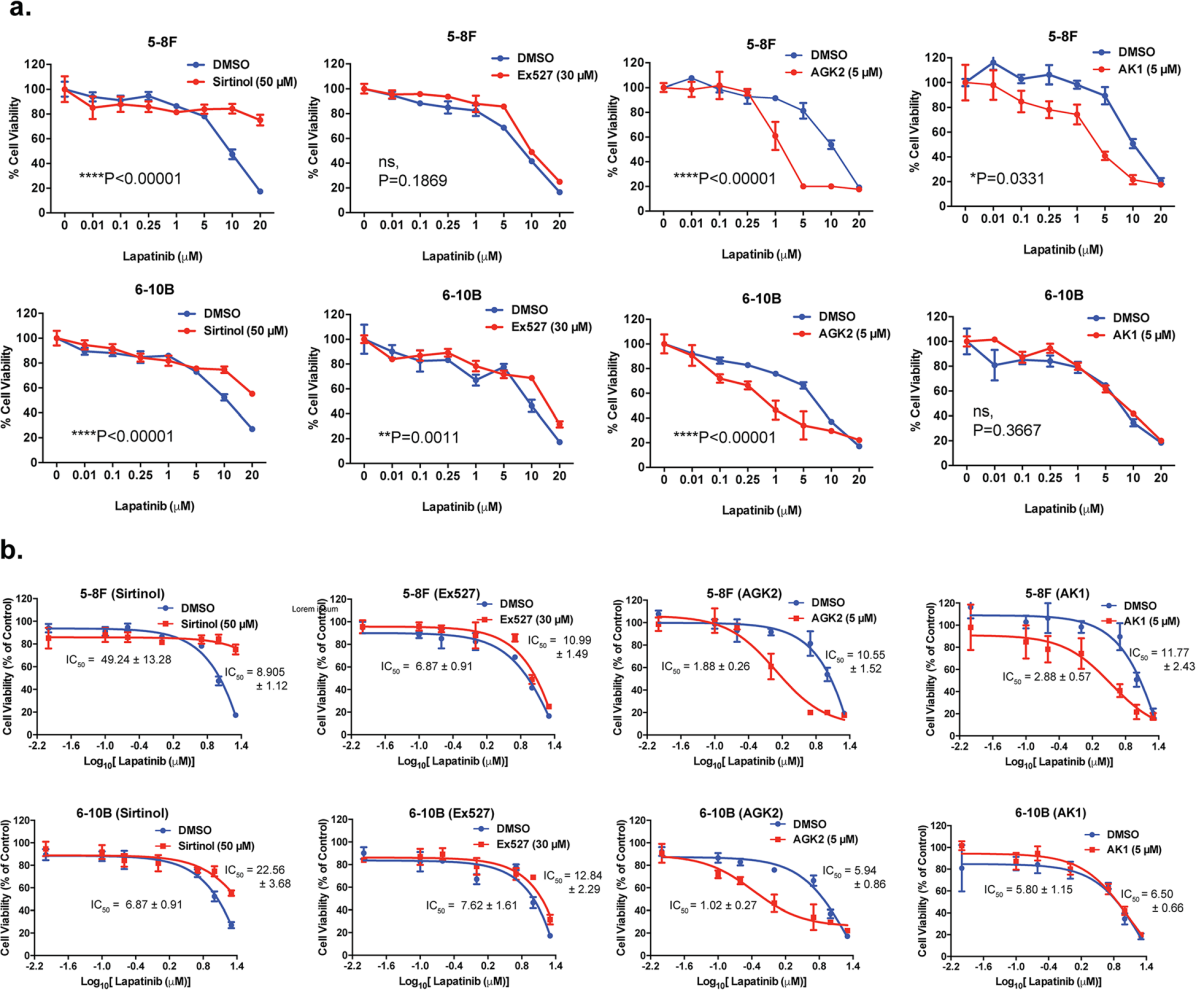
NPC cells show increased Lapatinib sensitivity when combined with AGK2 or AK1 but not Sirtinol or Ex527. **a** Cells (3 × 10^3^ / well) were seeded in 96-well plates and treated with increasing concentrations of Lapatinib for 48 h before staining and measurement of optical density at 492 nm. Data were analysed by generating curves using Graph-pad Prism. **b** Best-fit curves were generated to determine the effect of AGK2 or AK1 on reducing the IC_50_ of Lapatinib for each NPC cells. Data represent the average ± SEM of three different experiments (**** *P* < 0.0001)

Next, we tested if the SIRT inhibitors can act cooperatively with Lapatinib and enhance its antiproliferative functions. To this end, the resistant 5-8F and sensitive 6-10B NPC cells were cultured with sub-optimal levels of SIRT inhibitors (Fig. 2) or vehicle controls in the presence of increasing doses of Lapatinib and their cell viability examined by SRB assays (Fig. 5a). Surprisingly, combining the pan-SIRT inhibitor Sirtinol with Lapatinib significantly increased the overall viability of both NPC cell lines compared to Lapatinib treatment alone (2 way-ANOVA, ****P < 0.0001). Similarly, EX527 increased the viability of the Lapatinib-treated 6-10B cells (2 way-ANOVA, **P = 0.0011) but had no significant effects on the viability of the Lapatinib-treated 5-8F cells (2 way-ANOVA, non-significant, *P* = 0.3667). By contrast, the SIRT2 inhibitor AGK2 demonstrated additive effects on the cytotoxicity of Lapatinib (2 way-ANOVA, ****P < 0.0001, respectively) in both the sensitive 6-10B (IC_50_: 1.88 ± 0.26 to 5.94 ± 0.86 μM) and the resistant 5-8F (10.55 ± 1.52 to 1.88 ± 0.26 μM) NPC lines (Fig. 5b). Similarly, the SIRT2-inhibitor AK1 also functioned additively with Lapatinib in the 5-8F cells (2 way-ANOVA, *P = 0.0331, IC_50_, 11.77 ± 2.43 to 2.88 ± 0.57 μM) but had no significant positive effects on Lapatinib in the sensitive 6-10F cells (2 way-ANOVA, non-significant P = 0.3667, IC_50_, 5.80 ± 1.15 to = 6.50 ± 0.66 μM). AK1 did not demonstrate any additive effects with Lapatinib in 6-10B cells, probably because of the predominant anti-proliferative function of Lapatinib in the Lapatinib-sensitive cells. Moreover, Lapatinib also functions to downregulate SIRT2 expression in 6-10B, and therefore, AK1 might be redundant in inhibiting SIRT2 after the downregulation of SIRT2 in these cells by Lapatinib. However, overall the SIRT2 inhibitors AGK2 and AK1, but not the SIRT1 and pan-SIRT inhibitors, demonstrated additive antiproliferative effects on Lapatinib in SRB cell proliferative assays, suggesting that SIRT2 plays a specific role in limiting Lapatinib cytotoxicity and in promoting Lapatinib resistance in NPC cells.

**Fig. 6:**
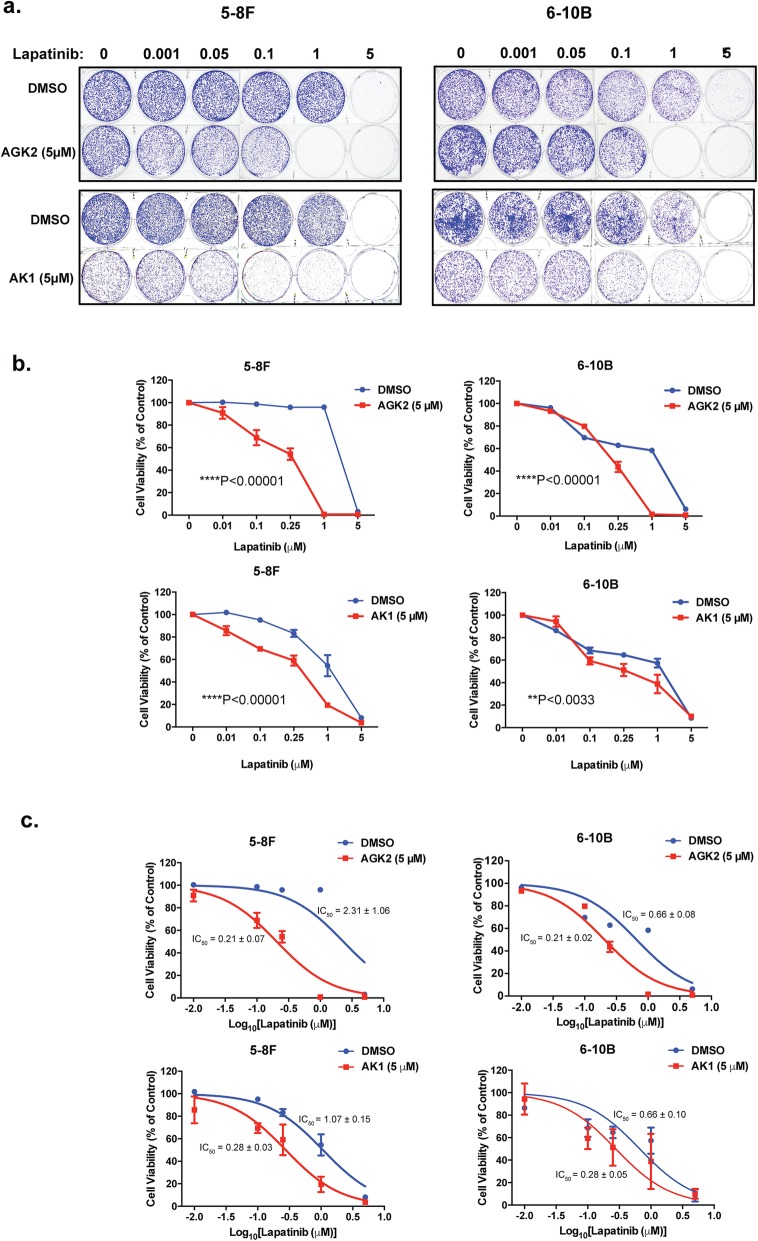
Lapatinib in combination with AGK2/AK1 increase long term cell sensitivity compared to Lapatinib alone. **a** 5-8F and 6-10B cells were seeded in 6-well plates and treated with the Lapatinib concentration indicated for 72 h. Five micromolar AGK2 and AK1 was used for combination treatment. Cell culture media was then removed and colony formation observed for 15 days. At the end of this time period, cells were fixed with 4% paraformaldehyde and stained with crystal violet. Representative pictures from triplicate experiments are shown. **b** The stain was solubilised with 33% acetic acid and absorbance at 592 nm were obtained. Graphs were generated to show the effect of combination treatment. **c** Best fit-curves were generated to determine the effect of AGK2/AK1 on the IC_50_ of Lapatinib in 5-8F and 6-10B cells.

To ascertain further the role of SIRT2 in enhancing Lapatinib resistance in NPC cells, we performed clonogenic survival assays on the two NPC cell lines to study the long-term antiproliferative effects of the two SIRT2 inhibitors AGK2 and AK1 on Lapatinib (Fig. 6a). In clonogenic assays, both SIRT2-inhibitors displayed additive effects on Lapatinib in NPC cell lines (2 way-ANOVA, ****P < 0.0001, for all except **P < 0.0033 for AK1 in 6-10B), further reducing the overall clonogenic survival of Lapatinib-treated cells (Fig. 6b). When combined with Lapatinib, AGK2 showed additive effects on the anti-proliferative functions of Lapatinib in both the Lapatinib resistant 5-8F (IC_50_: 2.31 ± 1.06 to 0.21 ± 0.07 μM) and sensitive 6-10B (IC_50_: 0.66 ± 0.08 to 0.21 ± 0.02 μM) NPC cells (Fig. 6c). The SIRT2-inhibitor AK1 also functioned additively with Lapatinib in both the resistant 5-8F and (IC_50_: 1.07 ± 0.15 to 0.28 ± 0.03 μM) and sensitive 6-10B (IC_50_: 0.66 ± 0.10 to 0.28 ± 0.05 μM) cells (Fig. 6c).

### Silencing of SIRT2 enhances Lapatinib sensitivity of NPC cells

**Fig. 7:**
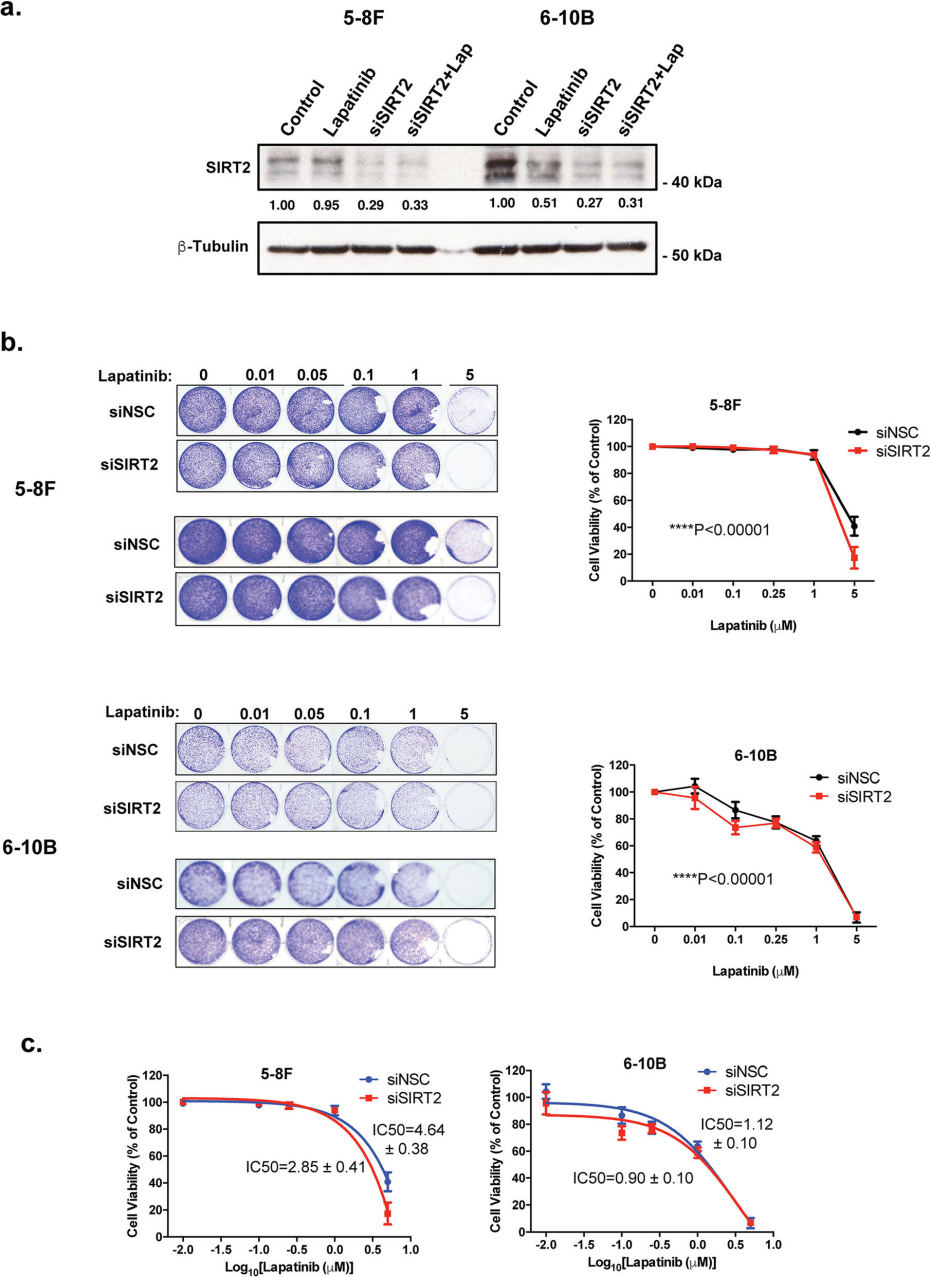
Silencing of SIRT2 increases long-term Lapatinib cytotoxicity in NPC cells. **a** NPC cells were transfected with siNSC and siSIRT2 smartpools. After 24 h both siNSC and siSIRT2-transfected cells were treated with DMSO or 5 μM Lapatinib for another 24 h. Forty-eight h after transfection, cells were harvested and SIRT2 protein knockdown was confirmed by western blot analysis. Protein levels were determined by using ImageJ and the protein levels relative to β-tubulin are shown below protein bands as ratios to the 0 h Lapatinib controls. **b** Forty-eight h after transfection, 1000 cells were seeded per well, into 6-well plates and then treated with the Lapatinib concentrations of 0, 0.01, 0.05, 0.1, 1 and 5 μM for 72 h and colony formation observed for 15 days. At the end of this time period, cells were fixed with 4% paraformaldehyde and stained with crystal violet. The stain was solubilised with 33% acetic acid and absorbance at 592 nm were obtained. Graphs were generated to show the effect of silencing SIRT2 on Lapatinib sensitivity. **c** Best fit-curves were generated to determine the effect of SIRT2 knock down on the IC_50_ of Lapatinib in 5-8F and 6-10B cells. Numerical data represent the average ± SEM of three different experiments (**** *P* < 0.0001)

To confirm further the role of SIRT2 in promoting Lapatinib resistance, we next examined if siRNA-depletion of SIRT2 can enhance the antiproliferative effects of Lapatinib in the 5-8F and 6-10B NPC cells using clonogenic assays. Knock-down of SIRT2 was confirmed by western blotting analysis before the NPC cells were subjects to clonogenic assays (Fig. 7a). The results showed that despite the subtle effects, depleting SIRT2 has additive effects on Lapatinib when compared with the non-silencing siRNA controls in both the NPC cell lines (2 way-ANOVA, ****P < 0.0001, respectively) (Fig. 7b). Significantly, the SIRT2-siRNA enhanced the anti-proliferative functions of Lapatinib in both the resistant 5-8F and (IC_50_: 4.64 ± 0.38 to 2.85 ± 0.41 μM) and sensitive 6-10B (IC_50_: 1.12 ± 0.10 to 0.90 ± 0.10 μM) cells (Fig. 7c). In consequence, the results suggest that SIRT2 knockdown can significantly enhance the cytotoxic and cytostatic functions of Lapatinib in both the sensitive and resistant NPC cells, consistent with the findings from the SIRT2 pharmaceutical inhibitors, AK1 and AGK2. Collectively, these results demonstrated that selective inhibition of SIRT2 can promote Lapatinib sensitivity, indicating SIRT2 is a potential target for overcoming Lapatinib resistance in NPCs.

### Inhibition and silencing of SIRT2 can combine with Lapatinib to promote FOXO3 acetylation

**Fig. 8:**
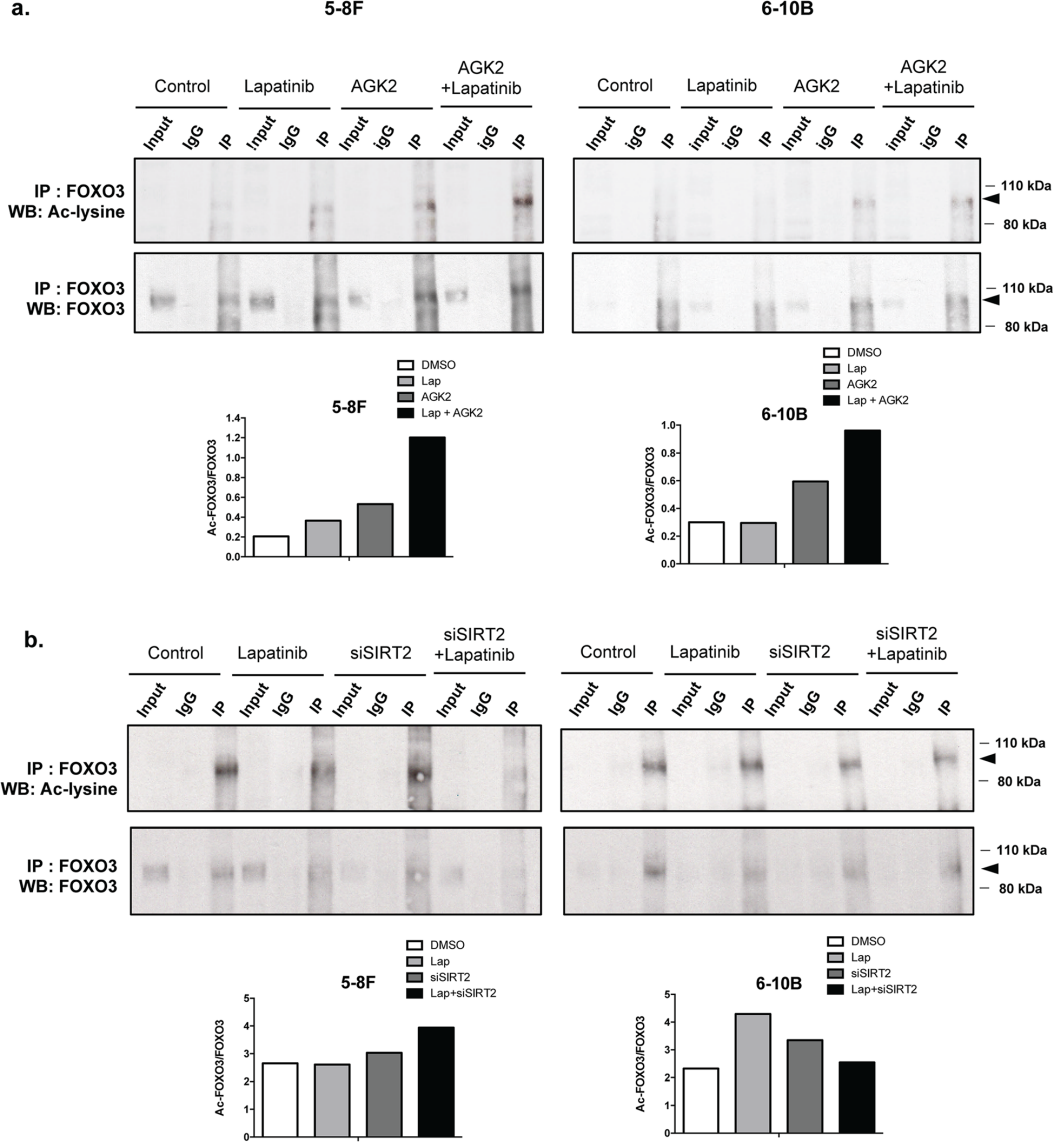
Inhibition of SIRT2 by AGK2 as well as silencing of SIRT2 in combination with Lapatinib upregulates acetylated FOXO3 levels in 5-8F and 6-10B cells. **a** NPC were treated with AGK2 alone or in combination with Lapatinib for 48 h. Proteins were obtained from whole cell extracts and assessed by co-immunoprecipitation (co-IP) with an anti-FOXO3 antibody. Subsequent immunoblotting was performed using antibodies against Ac-Lys and FOXO3. The anti-IgG was used as a negative control. Acetylated FOXO3 levels were determined by using ImageJ analysis and representative bar diagram are shown (bottom panel after the blot). **b** NPC cells were transiently transfected with siNSC and siSIRT2 and treated with 5 μM Lapatinib for 24 h. Proteins were obtained from whole cell extracts and assessed by Co-immunoprecipitation (Co-IP) with an anti-FOXO3 antibody. Subsequent immunoblotting was performed using antibodies against Ac-Lys and FOXO3. Acetylated and total FOXO3 levels were determined by using ImageJ analysis and representative bar diagrams demonstrating relative acetylated to total FOXO3 ratios are shown (bottom panel after the blot).

To confirm that SIRT2 can modulate the ability of Lapatinib to mediate FOXO3 acetylation, we next determined if chemical inhibition or silencing of SIRT2 can affect FOXO3 acetylation in the absence or presence of Lapatinib treatment using co-immunoprecipitation (IP) assays (Fig. 8). Relative FOXO3 acetylation levels were then determined by comparing the ratio of acetylated to total FOXO3 co-precipitated. The computed results showed that SIRT2-depletion by siRNA or -inhibition by AGK2 can induce FOXO3 acetylation in both the Lapatinib resistant 5-8F and the sensitive 6-10B cell lines (Fig. 8a and b). The chemical inhibitor AGK2 is more effective in inducing FOXO3 acetylation than SIRT2 siRNA probably because it is more effective in inhibiting SIRT2 activity. Importantly, both chemical inhibition and siRNA-depletion of SIRT2 enhanced the FOXO3 acetylation induced by Lapatinib in both the Lapatinib resistant 5-8F and sensitive 6-10B cell lines, except for the 6-10B cells treated with SIRT2 siRNA. The low levels of acetylated FOXO3 precipitated from the sensitive 6-10B following combined Lapatinib and SIRT2-siRNA treatment is likely to be due to the high levels of global protein degradation as a result of cell death caused by the combination of SIRT2-depletion and Lapatinib treatment. Moreover, Lapatinib also downregulates SIRT2 in the sensitive 6-10B cell line, rendering the effects of SIRT2-siRNA redundant. Overall, these IP results suggest that SIRT2 restricts the Lapatinib-mediated FOXO3 acetylation in NPCs. In summary, our collective results reveal that SIRT2 has a role in modulating FOXO3 acetylation and Lapatinib response in NPCs and that targeting SIRT2 can enhance the sensitivity of NPCs to Lapatinib and to overcome Lapatinib-resistance in NPC.

## Discussion

Metastasis and drug resistance are the characteristics of aggressive cancers and the major causes for poor survival in nasopharyngeal carcinoma (NPC) [12, 13]. An understanding of the mechanisms involved in the development of NPC metastasis and drug resistance will aid the development of early diagnostic biomarkers and the identification of potential therapeutic targets. Previous studies have reported Lapatinib could effectively induce cell death and autophagy in NPC cells [12, 13]. Clinical studies also suggest that Lapatinib alone or combined with standard chemotherapies are well-tolerated and promote patient survival in metastatic/recurrent head and neck squamous cell carcinoma [28]; however, not all patients benefit from Lapatinib-based treatments [29, 30]. In the present study, we found that the highly metastatic (C666–1 and 5-8F) NPC lines are significantly more Lapatinib resistant compared to poorly metastatic lines (6-10B, TW01 and HK-1), suggesting that the molecular mechanisms involved in metastasis in NPC may overlap with those responsible for the development of Lapatinib resistance.

FOXO3 is a tumour suppressive transcription factor and an important modulator of sensitivity to chemotherapy [31, 32]. FOXO3 inhibits cell growth by driving the transcription of genes, such as Bim, FasL, p27^Kip1^, p130 (RB2), essential for cell proliferative arrest, cell death and differentiation [31, 32]. Conversely, inactivation of FOXO3 is a crucial step for oncogenic transformation and the development of cytotoxic drug resistance [31, 32]. Previous work has also suggested that FOXO3 mediates the cytotoxicity of Lapatinib in breast cancer [33, 34]. In here, we have shown definitively using foxo1/3/4-deficient fibroblasts that FOXO3 along with FOXO1 and − 4 are involved in mediating the cytotoxic functions of Lapatinib. Consistent with this, recent evidence has showed that natural products, such as curcumin, inhibits the growth and induces apoptosis in NPC cell by increasing FOXO3 expression [35]. In addition, the G-quadruplex ligand SYUIQ-5 also induces NPC autophagy by down-regulating Akt phosphorylation and promoting FOXO3 nuclear translocation [36].

The activity, expression and subcellular localization of FOXO3 are regulated by a diverse range of post-translational modifications [37]. Phosphorylation by kinases, particularly Akt (also called PKB) FOXO3, ERK, IKB kinase (IKK) and serum and glucocorticoid-regulated kinase (SGK) can enhance FOXO3 nuclear to cytoplasmic shuttling and its degradation [32, 37]. Conversely, other kinases, such as p38 MAPK [38], stress activated c-Jun-NH2-kinase (JNK) [39] have also been demonstrated to promote FOXO3 activity and expression. Consistent with previous studies with EGFR1/HER2(ERBB2)-targeted tyrosine kinase inhibitors (TKIs) [34, 40], we found that Lapatinib can cause FOXO3-dephosphorylation (T32) and the downregulation of its target FOXM1 in Lapatinib-sensitive NPC cells. However, although EGFR1/HER2 inhibitors have previously been shown to modulate p38 and JNK activity [41, 42], we did not observe any substantial changes in p38 and JNK phosphorylation/activity in NPC cells in response to Lapatinib treatment, suggesting it is unlikely that Lapatinib modulates FOXO3 activity via p38 and JNK in these NPC cells. FOXM1 is a potent oncogene negatively regulated by FOXO3 [32] and contributes to cancer drug resistance through controlling many genes involved in cell proliferation, survival, DNA repair, and tubulin destabilization [32, 43, 44]. Consistent with a role for FOXO3 in Lapatinib action in NPC cells, we found that FOXM1 expression is repressed by Lapatinib in the sensitive cells but remains constitutively high in the resistant NPC cells, suggesting a role of FOXM1 in NPC Lapatinib resistance. In agreement, FOXM1 has been shown to be able to mediate paclitaxel resistance by regulating the gene transcription of the ABCC5 drug efflux transporter in NPC [18]. In addition, overexpression of FOXM1 is directly associated with metastasis in NPC, and targeting FOXM1 with inhibitors or siRNA knockdown can effectively restrict the cell proliferation, migration, angiogenesis and survival of NPC cells [29, 45].

Besides phosphorylation, FOXO3 is also regulated by other post-translational modifications such as acetylation, methylation, ubiquitination and glycosylation (Zhao et al., 2011). The nuclear sirtuins SIRT1, SIRT2 and SIRT6 have been shown to negatively regulate FOXO3 acetylation and its activity [46–48]. In the present study, we found that SIRT2 specifically modulates the cytotoxicity of Lapatinib and is linked to Lapatinib resistance using specific chemical inhibitors and SIRT2 siRNAs. Although our findings show that all four SIRT inhibitors (i.e. sirtinol, EX527, AGK2 and AK1) can limit NPC cell proliferation, only the SIRT2 specific inhibitors AGK2 and AK1 function cooperatively with Lapatinib, supporting the idea that SIRT2 specifically modulates Lapatinib response and resistance. Nevertheless, all four SIRT inhibitors can restrict normal NPC cell viability and clonogenicity, and this suggests that the nuclear sirtuins SIRT1, − 2 and − 6 detected in NPC are all potential oncogenes. In agreement, SIRT1 upregulation has been shown to be associated with tumour progression and metastasis in NPC biopsies [49]. The pan-SIRT inhibitor sirtinol has previously been shown to induce p53 acetylation and cell death through targeting both SIRT1 and SIRT2 [50]. However, like EX527, the sirtinol concentrations employed here preferentially inhibit SIRT1 and have limited activities towards SIRT2 [50]. Therefore, these observations support further the notion that SIRT2 specifically moderates the cytotoxic action of Lapatinib and mediates Lapatinib resistance, independently of SIRT1. The reason for the finding that both the SIRT inhibitors sirtinol and EX527 oppose the cytotoxic functions of Lapatinib is unclear. However, a context dependent tumour suppressive role for SIRT1 has previously been proposed [26]. In concordance, SIRT1-deficient cells have been shown to be defective in the ability to normally upregulate the p19(ARF) senescence mediator and its potent downstream tumour suppressor p53 [26], and this might account for the ability of sirtinol and EX527 to attenuate the antiproliferative function of Lapatinib. Inhibition of SIRT2 by chemical inhibitor or silencing significantly enhances the cytotoxicity of Lapatinib not only in the sensitive but also in the resistant NPC cells, suggesting that SIRT2 not only modulates the cytotoxic functions of Lapatinib in the sensitive NPC cells, but it also mediates Lapatinib resistance. This implies that targeting SIRT2 can enhance the cytotoxicity of Lapatinib and may represent a novel strategy for overcoming Lapatinib resistance in NPC. This role of SIRT2 in modulating Lapatinib response and sensitivity is confirmed by SIRT2 depletion experiments showing silencing SIRT2 can enhance the cytotoxicity in both Lapatinib sensitive and resistant NPC cells. Although the siRNA depletion approach has less off-target effects, it also relies on high delivery efficiencies; the incomplete SIRT2 depletion might account for the small additional effects on Lapatinib.

In agreement, overexpression of SIRT2 has been shown to essentially lengthen the M phase and defer mitotic exit [15]. Moreover, SIRT2 can induce the cell proliferation of leukaemia and resistance to apoptosis [51]. Conversely, decreased SIRT2 activity can reduce glioma cell survival by induced both necrosis and apoptosis [52] and limit melanoma cell growth and clonogenicity [53]. Furthermore, overexpression of SIRT1 and SIRT2 can also confer resistance to chemotherapy such as paclitaxel [54].

Furthermore, our co-IP experiments also show that the Lapatinib induces FOXO3 acetylation and that inhibition of SIRT2 by chemical inhibitor or silencing also promotes FOXO3 acetylation in NPC cells. Importantly, SIRT2 inhibition or silencing can combine with Lapatinib to cause further enhancement of FOXO3 acetylation than Lapatinib treatment alone in both sensitive and resistant NPC cells, suggesting that SIRT2 can moderate the cytotoxic functions of Lapatinib and promote resistance through limiting FOXO3 acetylation. In agreement, FOXO3 deacetylation by SIRT2 has previously been reported to enhance FOXO3 ubiquitination and proteasomal degradation [55].

## Conclusion

Collectively, these data suggest that the cytotoxic functions of Lapatinib are mediated through the acetylation and activation of FOXO3, and that SIRT2 can specifically antagonise the cytotoxicity of Lapatinib through mediating FOXO3 deacetylation in both sensitive and resistant NPC cells. The present findings also suggest that SIRT2 can be an important biomarker for metastatic and Lapatinib resistant NPC and that targeting the SIRT2-FOXO3 axis may provide a means to treat NPC and to overcome NPC chemoresistance.

## Data Availability

Data sharing is not applicable to this article as no datasets were generated or analysed during the current study.

## Abbreviations

EGFR: Epidermal growth factor receptor
FOX: Forkhead box
NPC: Nasopharyngeal carcinoma
SIRT2: Sirtuin-2
